# Self-Discharge of a Proton Exchange Membrane Electrolyzer: Investigation for Modeling Purposes

**DOI:** 10.3390/membranes11060379

**Published:** 2021-05-22

**Authors:** Ángel Hernández-Gómez, Victor Ramirez, Damien Guilbert, Belem Saldivar

**Affiliations:** 1 Department of Renewable Energy, Centro de Investigación Científica de Yucatán (CICY), Mérida P.C. 97205, Mexico; angel.hernandez@cicy.mx (Á.H.-G.); victor.ramirez@cicy.mx (V.R.); 2 Cátedras CONACYT, Ciudad de México P.C. 03940, Mexico; belemsaldivar@hotmail.com; 3 Group of Research in Electrical Engineering of Nancy (GREEN), Université de Lorraine, GREEN, F-54000 Nancy, France; 4 Facultad de Ingeniería, Universidad Autónoma del Estado de México (UAEM), Ciudad de México, Toluca P.C. 50000, Mexico

**Keywords:** PEM electrolyzer, modeling, dynamic model, self-discharge voltage

## Abstract

The self-discharge phenomenon results in a decrease of the open-circuit voltage (OCV), which occurs when an electrochemical device is disconnected from the power source. Although the self-discharge phenomenon has widely been investigated for energy storage devices such as batteries and supercapacitors, no previous works have been reported in the literature about this phenomenon for electrolyzers. For this reason, this work is mainly focused on investigating the self-discharge voltage that occurs in a proton exchange membrane (PEM) electrolyzer. To investigate this voltage drop for modeling purposes, experiments have been performed on a commercial PEM electrolyzer to analyze the decrease in the OCV. One model was developed based on different tests carried out on a commercial-400 W PEM electrolyzer for the self-discharge voltage. The proposed model has been compared with the experimental data to assess its effectiveness in modeling the self-discharge phenomenon. Thus, by taking into account this voltage drop in the modeling, simulations with a higher degree of reliability were obtained when predicting the behavior of PEM electrolyzers.

## 1. Introduction

Electrolyzers are reliable devices that have been employed for various applications in residential, commercial, and industrial areas thanks to their well-established technology [1,2]. Additionally, electrolyzers do not require continuous maintenance since they hardly include mobile elements [3]. The main function of electrolyzers is to produce high-purity hydrogen (up to 99.999 vol %) by using the water electrolysis process, so the produced hydrogen can be directly used in low-temperature fuel cells, which are sensitive to impurities of the hydrogen stream [4]. Currently, according to the type of electrolyte, there are three main technologies: proton exchange membrane (PEM) electrolyzer, alkaline electrolyzer, and solid oxide (SO) electrolyzer.

Although each type of electrolyzer has its advantages and disadvantages, PEM electrolyzers have attracted a lot of attention in recent years from researchers due to their compact system design, specific production capacity, high energy efficiency, and simplicity [5,6]. Additionally, compared to alkaline technology, PEM technology provides superior performance when it is coupled with renewable energy sources (RES) due to its high flexibility and quick responses to dynamics. This important feature enables capturing energy during dynamic operations, which are consistent during the operation of RES. On the other hand, since electrolyzers are electrochemical devices such as batteries and supercapacitors, they feature a self-discharge phenomenon. This phenomenon is caused by internal chemical reactions leading to a decrease of the open-circuit voltage (OCV) when the electrolyzer is disconnected from the power supply. In comparison, for batteries and supercapacitors, the self-discharge phenomenon reduces their stored charge and their lifespan.

To disseminate PEM electrolyzer technology on a large-scale market, different research works have been carried out. In particular, mathematical modeling is a valuable tool for predicting static and dynamic behavior when coupling PEM electrolyzers with RES [7,8]. Research using modeling tools has been of vital importance to studying the electrical domain in PEM electrolyzers, which is the key to understanding and enhancing the performance of the electrolyzer [9,10]. Besides, different models have already been proposed and can be divided into three categories. The first category concerns empirical models reported in [6,9]. These models allow taking into consideration the electrical domain of PEM electrolyzers, including the OCV, activation, ohmic, and concentration overvoltage according to the temperature and pressure. These models are useful for developing electrolyzer emulators [6]. In comparison, the second category includes semi-empirical models proposed in [11,12]. The voltage–current curve describing the performance of the electrolyzer is modeled by using semi-empirical equations depending on the temperature and pressure. Both developed semi-empirical and empirical models neglect the dynamic operations of electrolyzer, and they have to be taken into consideration for coupling the electrolyzers with renewable energy sources. As a result, the last category is related to dynamic models firstly developed in [13,14]. The dynamic behavior and the losses of the electrolyzer have been modeled as an equivalent electrical circuit whose parameters strongly depend on the operating conditions (supply current, temperature, and pressure). Additionally, investigations on how voltage affects other electrolyzer parameters have been reported in the literature. For example, in [15], the authors have investigated the gas crossover phenomenon, which is related to the ohmic loss of the membrane. Other important parameters are the efficiencies in the PEM electrolyzer, which indicate the performance of the device (i.e., if the specific energy consumption regarding the hydrogen production is efficient) [16]. The main efficiencies reported in the literature are Faraday’s efficiency, voltage efficiency, and energy efficiency [16,17].

However, despite these extensive research on PEM electrolyzer modeling, no work in the literature has reported the phenomenon of self-discharge voltage in PEM electrolyzers. The term "self-discharge" is sometimes associated with the chemical reactions discharging the surface and excluding any physical processes which cause the voltage drop [18,19]. The phenomenon of self-discharge voltage has proven to be an important subject of study for any electrochemical device, particularly for batteries and supercapacitors. For batteries, the self-discharge voltage is the main limitation to storing energy for a long time, so the efficiency and autonomy of systems are poor [20], which is a challenging issue when using batteries in electric vehicles, industries, and residences [21,22]. To reduce battery self-discharge voltage, many strategies have been developed, such as optimizing materials for battery construction [22,23] and developing different models with a high degree of reliability (i.e., by taking into account several variables or assimilating the battery like an electronic circuit) [24,25]. On the other hand, for supercapacitors, besides being determinant for the duration of energy storage (i.e., rest phases), self-discharge voltage is an important indicator to quantify performance [26,27]. As a result, by decreasing the effects of supercapacitors’ self-discharge voltage, the lifespans of devices that depend on the power supply of supercapacitors are improved [28,29]. Like batteries, different materials for the construction of supercapacitors have been used, and the development of models has been implemented as a strategy to decrease the self-discharge voltage [30,31].

In comparison, the self-discharge phenomenon for fuel cells is usually neglected in the literature, and the first investigation about this important issue was reported in [32]. It has been highlighted that decreases of the OCV of fuel cells are mainly caused by the leakage currents due to gas crossover through the membrane. On the one hand, currently manufactured membranes feature thin membranes to reduce the ohmic losses associated with the conduction of protons. On the other hand, the thinner the membrane, the higher the gas crossover and leakage currents [33,34]. Over a long period of operation, gas crossover through the membrane may reduce the lifespan of the fuel cell and degrade the membrane (e.g., pinholes) [34]. Therefore, this phenomenon remains a major concern for electrolyzers and further investigations are required.

From this current state-of-the-art, it is important to start studying the phenomenon of self-discharge voltage that occurs in PEM electrolyzers, since it is significant when the electrolyzer is coupled with RES and should be useful for the development of future works as PEM electrolyzer emulators (i.e., by taking this phenomenon into account, more realistic behavior of the PEM electrolyzer voltage can be obtained). For this reason, this we aimed at describing the self-discharge voltage met in a PEM electrolyzer through different experimental tests and developing a model enabling reproducing the self-discharge phenomenon according to the operating conditions, such as the current. This model has been developed based on models proposed in the literature for supercapacitors and batteries to describe the behavior of self-discharge voltage. The comparison between the developed model and the experimental data for different operating conditions has demonstrated the high reliability of the model in reproducing the self-discharge phenomenon.

This paper is organized as follows. After the introduction, the sighting of the self-discharge voltage in a PEM electrolyzer (description of the experimental test setup and self-discharge voltage issues) is described in Section 2. The development of the model for self-discharge voltage is introduced in Section 3. Validation and discussion are provided in Section 4. Finally, the conclusion of this work is presented in Section 5.

## 2. Sighting of the Self-Discharge Voltage in a PEM Electrolyzer

### 2.1. Description of the Experimental Test Setup

**Figure 1:**
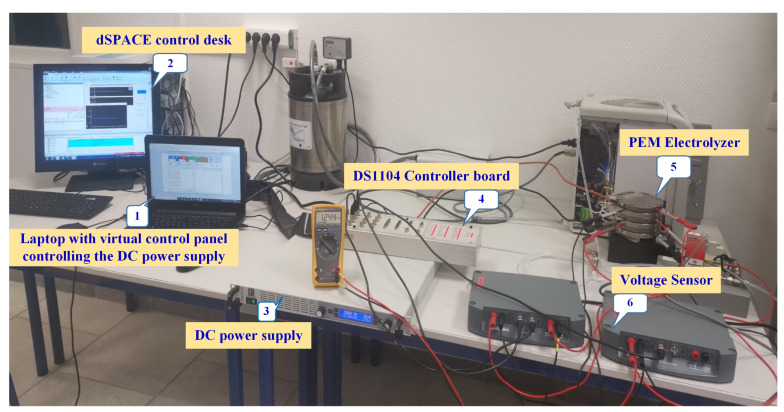
Experimental test setup.

To study the self-discharge voltage of the PEM electrolyzer stack, an experimental test rig has been designed and built at the GREEN laboratory, IUT de Longwy, as shown in Figure 1. The experimental test rig is composed of the following devices and components: (1) a laptop; (2) dSPACE control desk software; (3) a DC power supply; (4) a DS1104 controller board; (5) a commercial-400W PEM electrolyzer; (6) a voltage probe MTX 1032-B from the Metrix Company to acquire the stack voltage. The DC power supply is controlled through a virtual control panel installed on the laptop. The PEM electrolyzer is supplied from pure water featuring low water conductivity (less than 2 $\mu S\cdot m^{-1}$). Higher water conductivity can contaminate and damage the electrolyzer. The specifications of the studied commercial PEM electrolyzer system NMH2 1000 from HELIOCENTRIS*®* Company are provided in Table 1. Since the PEM electrolyzer NMH2 1000 system has been developed for educational purposes, it includes power electronics connected to the stack. In this work, only the stack of the PEM electrolyzer is considered and connected to an external DC power supply as depicted in Figure 1. The studied PEM electrolyzer has a solid polymer electrolyte based on fluoropolymer Nafion material from DuPont*®* company. The thickness of the membrane is very thin (around 25 μm), resulting in lower ohmic losses and may influence the self-discharge phenomenon based on previous works reported for PEM fuel cells [33,34] and electrolyzers [15,35]. The acquired stack voltage from the voltage probe is then transferred into the DS1104 controller board. Finally, the experimental data are monitored and saved through dSPACE control desk software. The sampling time was chosen as 1 s to collect the experiment data. The obtained experimental data were plotted by using Matlab-Simulink*®* software.

### 2.2. Self-Discharge Voltage Issues

**Figure 2:**
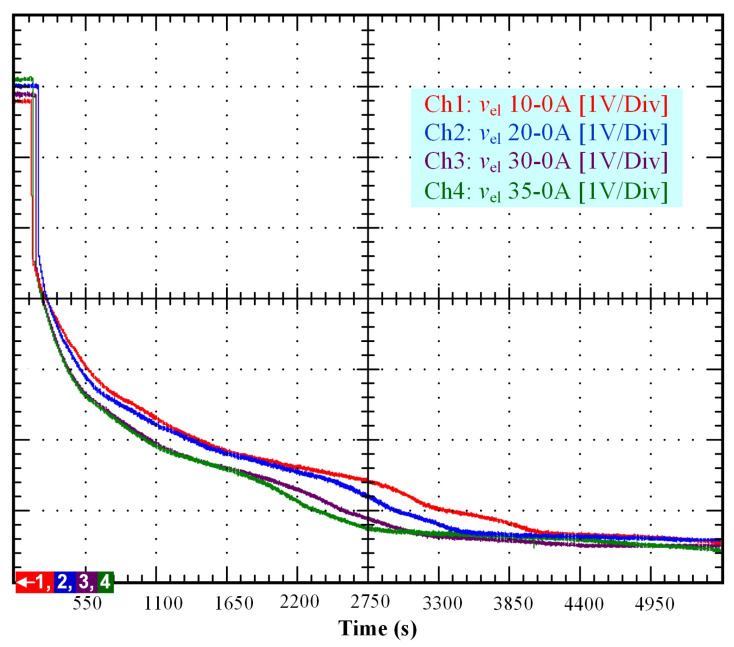
Self-discharge tests according to different initial conditions (10, 20, 30, and 35 A).

From the experimental test rig presented in Figure 1, several experimental tests based on different initial conditions (10, 20, 30, and 35 A) have been performed to emphasize the self-discharge phenomenon in a PEM electrolyzer. The obtained results are depicted in a single figure (Figure 2) to make easier their analysis for comparison purposes. The duration of the test was 5500 s. These tests consisted of supplying first the PEM electrolyzer with a constant current (10, 20, 30, and 35 A) for one hundred seconds, and then disconnecting the PEM electrolyzer from the DC power supply to observe the decrease of OCV. Based on Figure 2 it is important to point out that the duration in which a constant current is applied to the electrolyzer, and the initial operating conditions, influence the self-discharge phenomenon. As a result of the disconnection from the DC power supply, an immediate voltage drop can be observed in the stack voltage. This immediate decrease in the stack voltage can be explained based on a previous study where an equivalent electrical circuit was developed to model the static and dynamic behavior of the PEM electrolyzer [13]. The immediate voltage drop was due to the sum of the equivalent resistances modeling the anode, cathode, and electrolyte. This first voltage drop ended when the stack voltage reached 4.2 V related to the OCV of the electrolyzer stack.

**Figure 3:**
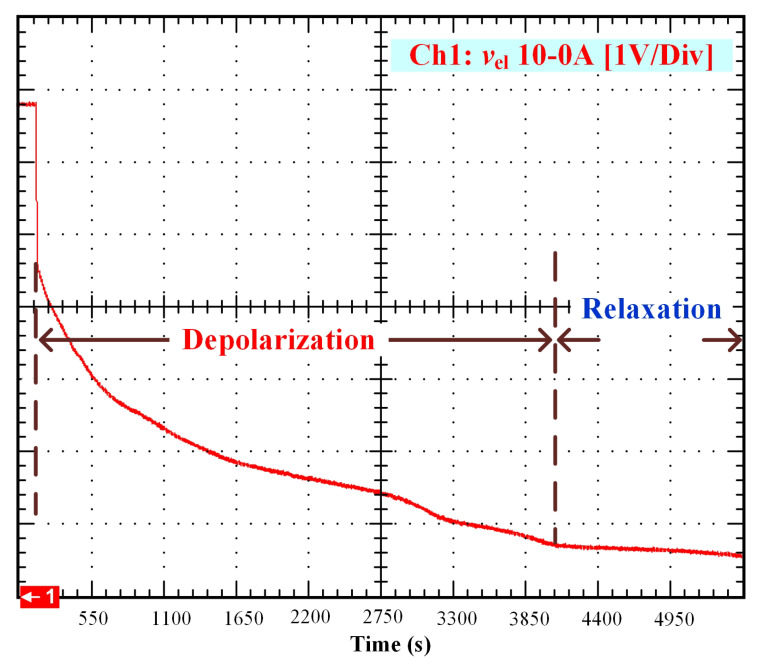
OCV depolarization and relaxation (10–0 A).

**Figure 4:**
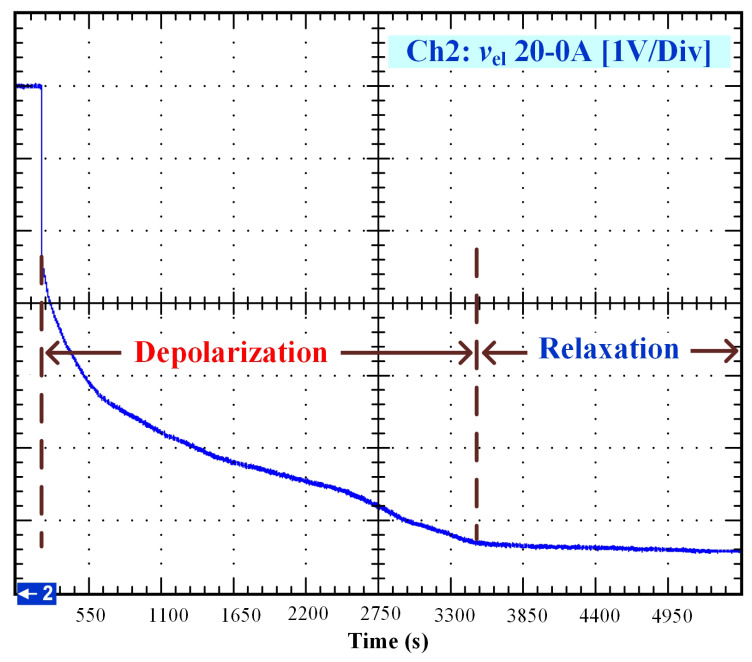
OCV depolarization and relaxation (20–0 A).

**Figure 5:**
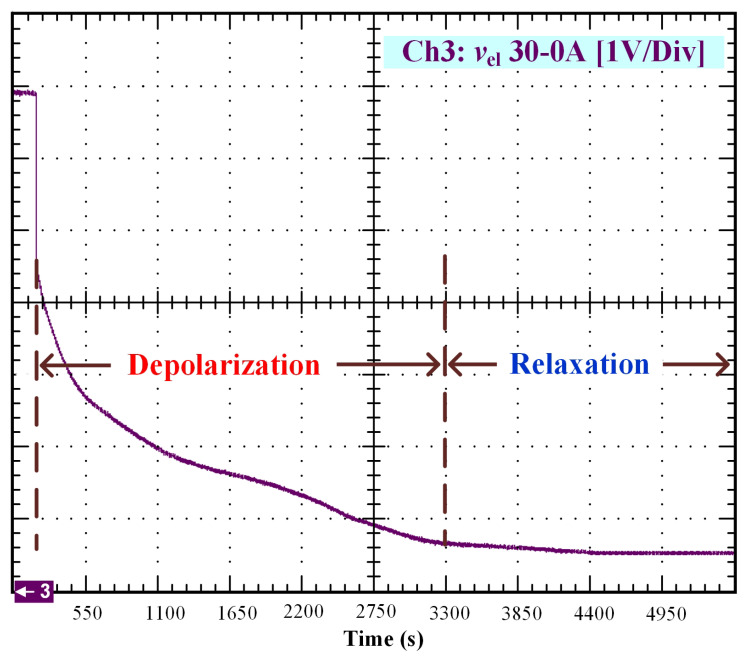
OCV depolarization and relaxation (30–0 A).

**Figure 6:**
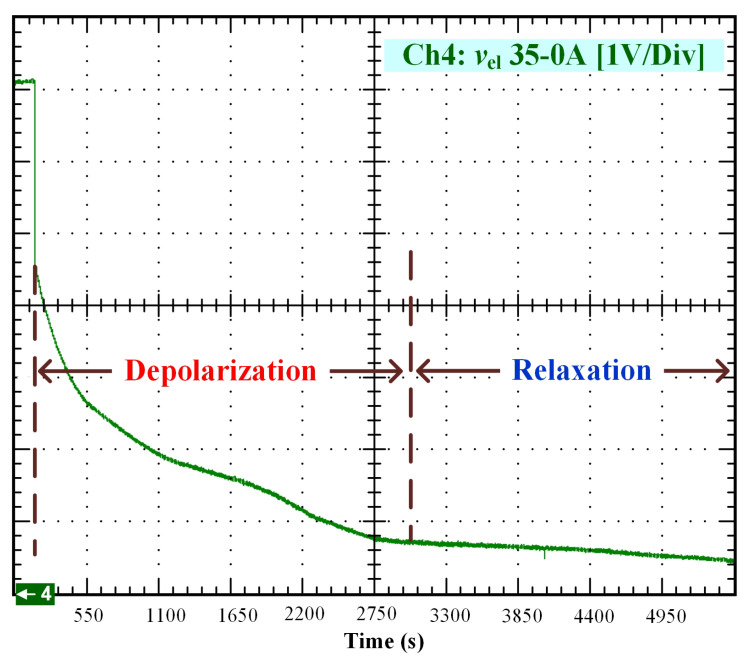
OCV depolarization and relaxation (35–0 A).

After that, the voltage drop related to the self-discharge phenomenon can be emphasized. This self-discharge can be divided into two phenomena: one is related to an accelerated self-discharge and the second to a slow, continuous self-discharge. These two phenomena are well known when characterizing batteries, as reported in the literature [36,37]. The accelerated self-discharge is commonly called OCV “depolarization,” and the slow continuous self-discharge is defined as OCV “relaxation”. In Figure 2, it can be seen that the OCV for the test (35–0 A) decreased more quickly and continuously compared to the other tests at lower currents. This observation demonstrates that the initial operating conditions affect the self-discharging. To emphasize those two phenomena (OCV depolarization and relaxation), the results of each test have been plotted in Figure 3, Figure 4, Figure 5 and Figure 6. The OCV relaxation occurred when the OCV reached around 0.5 V. For each test, the time separating these two OCV phenomena can be apprehended. Depending on the initial operating conditions, the duration of the OCV depolarization was longer or shorter. For instance, in Figure 6, the duration of the OCV depolarization is shorter (around 2850 s) compared to the other tests. A summary of the measured duration of the OCV depolarization for each test is provided in Table 2.

Based on the previous works reported for self-discharge in batteries [18,20] and supercapacitors [27,28], the studied PEM electrolyzer features a higher self-discharge rate. Indeed, lead-acid and lithium-ion batteries feature low self-discharge rates (noticeable in terms of days, weeks, or even months), and supercapacitors higher self-discharge rates (noticeable in terms of hours or days). In supercapacitors, the high self-discharge is due to their organic electrolyte. For batteries, the self-discharge rate strongly depends on the temperature and the wear (decrease in the number of charge/discharge cycles) due to deep discharges [18]. The higher the temperature, the higher the self-discharge rate. The same phenomenon has been reported when the batteries are subject to deep discharge. In comparison, for supercapacitors, it has been highlighted that their self-discharge rates are dependent on their double-layer capacitance values, and on the temperature [19]. The higher the temperature, the higher the self-discharge rate; whereas the smaller the double-layer capacitance value, the higher the self-discharge rate.

As it has been emphasized in previous works [13,14], when supplying the PEM electrolyzer with dynamic current profiles, the electrolyzer behaves as a capacitor because of the charge double-layer effect. Indeed, between the electrode and the electrolyte, there is a layer of charge, which can store electrical charge, and therefore, energy. The accumulation of charges generates an electrical voltage, which corresponds to the activation overvoltage both at the anode and at the cathode. Thus, when the current immediately changes, the activation overvoltage at both the anode and the cathode takes some time before following the change in current due to the reaction kinetics. Besides, the PEM electrolyzer features two dynamics, the first one slower regarding the anode, and the second one faster regarding the cathode. These two different dynamics can be modeled by two RC branches where their constant times depend on the values of the two resistors considering the values of both capacitances equal [38]. Given that the dynamics both at the anode and cathode change according to the operating conditions, the values of both RC branches cannot be considered constant. For this reason, in [14], the parameters of both RC branches have been assessed through mathematical modeling whatever the operating conditions. The reported values for the double-layer capacitance are similar to the ones for supercapacitors (between 3 and 69 F) [14]. By starting from this analysis and the previous observations reported for supercapacitors, the high-self discharge rate depicted in Figure 2 can be explained. Indeed, the low double-layer capacitance value of the PEM electrolyzer leads to an accelerated and slow continuous self-discharge.

Finally, based on previous works reported for PEM electrolyzers [15,35] and the knowledge of the authors on this topic, the self-discharge phenomenon in PEM electrolyzers is mainly induced by the leakage currents due to gas crossover through the membrane. Indeed, a part of the current at the cathode side has not been combined with the protons to generate hydrogen. Hence, this current goes back to the anode through the membrane that, at low current densities, is more permeable to gas crossover, as demonstrated in the literature [15,35]. Besides, it is important to point out that gas crossover is strongly dependent on temperature, pressure, and membrane thickness, as emphasized in the literature [15,39]. In the case under study, the PEM electrolyzer features a very thin membrane (i.e., around 25 μm). As highlighted in [39], the thinner the membrane, the higher the gas crossover. Therefore, the thin membrane of the PEM electrolyzer may explain the high self-discharge rate observed in Figure 2.

To conclude, despite these first observations, further investigation is required to understand the self-discharge rates of PEM electrolyzers and the parameters affecting the self-discharge rate, such as temperature and accelerated wear linked to their operating conditions (static and dynamic).

## 3. Mathematical Model

As mentioned in the introduction, there are no reported models for the self-discharge voltage of the PEM electrolyzer. For this reason, this work is also focused on developing a model to describe the self-discharge voltage behavior.

As can be seen, there is a lag time in the experimental data before self-discharge voltage occurs; see Figure 2. For this reason, it is assumed that the voltage in the electrolyzer has normal behavior before the self-discharge voltage occurs. Therefore, taking into account the model for voltage developed in [13,14], which describes the static-dynamic behavior of the voltage drop in small periods (i.e., time lapses around 50 s) into a PEM electrolyzer, the static-dynamic model for the voltage before the self-discharge voltage occurs can be expressed as:

(1) $$\begin{array}{c} V\left(t\right)=\left\{\begin{array}{cc} V_{0} & ift<t_{c},\\ V_{\mathrm{rev}}+i\left(t\right)\cdot R_{\mathrm{mem}}+\eta_{\mathrm{act}}\left(t\right) & ift\geq t_{c}, \end{array}\right. \end{array}$$

where $V_{0}$ is the initial voltage of the PEM electrolyzer (i.e., it is the value obtained before disconnecting the PEM electrolyzer from the power source). $V_{\mathrm{rev}}$ is the OCV, [V]. *i* is the current in the cell [A]; $R_{\mathrm{mem}}$ is the membrane resistance [Ω]. $\eta_{\mathrm{act}}$ is the activation overvoltage, [V]. *t* is the time in [s]. Finally, $t_{c}$ is the time that the electrolyzer remains connected to the energy source, [s].

Furthermore, due to the experiment carried out, the current function can be expressed as the step function:

(2) $$\begin{array}{c} i\left(t\right)=\left\{\begin{array}{cc} A_{1} & ift<t_{c},\\ A_{2} & ift\geq t_{c}, \end{array}\right. \end{array}$$

where both $A_{1}$ and $A_{2}$ take constant values, [A]. Besides, the activation overvoltage is expressed as $\eta_{act}=\eta_{act,c}+\eta_{act,a}$, where $\eta_{act,c}$ is:

(3) $$\begin{array}{c} \eta_{\mathrm{act},c}\left(t\right)=\left(\frac{\tau_{c}}{C_{c}}\right)\cdot \left[\left(A_{2}-A_{1}\right)\cdot \left(1-\exp\left[\frac{t_{c}-t}{\tau_{c}}\right]\right)+f_{1}\left(t\right)\right]+g_{1}\left(t\right) \end{array}$$

and $\eta_{\mathrm{act},a}$:

(4) $$\begin{array}{c} \eta_{\mathrm{act},a}\left(t\right)=\left(\frac{\tau_{a}}{C_{a}}\right)\cdot \left[\left(A_{2}-A_{1}\right)\cdot \left(1-\exp\left[\frac{t_{c}-t}{\tau_{a}}\right]\right)+f_{2}\left(t\right)\right]+g_{2}\left(t\right), \end{array}$$

where $C_{c}$ and $C_{a}$ are capacitance for cathode and anode; as for [F], both capacitances are considered equal based on a previous work reported in [38]. $\tau_{c}$ and $\tau_{a}$ are time constants, [s]. The functions $f_{1}\left(t\right)$ and $f_{2}\left(t\right)$ are given by:

(5) $$\begin{array}{c} \begin{array}{ccc} f_{1}\left(t\right)=A_{1}\cdot \left(1-\exp\left[\frac{-t}{\tau_{c}}\right]\right), & \; & f_{2}\left(t\right)=A_{1}\cdot \left(1-\exp\left[\frac{-t}{\tau_{a}}\right]\right), \end{array} \end{array}$$

and the functions $g_{1}\left(t\right)$ and $g_{2}\left(t\right)$ as:

(6) $$\begin{array}{c} \begin{array}{ccc} g_{1}\left(t\right)=\eta_{\mathrm{act},c}\left(0\right)\cdot \exp\left[\frac{-t}{\tau_{c}}\right], & \; & g_{2}\left(t\right)=\eta_{\mathrm{act},a}\left(0\right)\cdot \exp\left[\frac{-t}{\tau_{a}}\right]. \end{array} \end{array}$$

To model the self-discharge voltage, the models of supercapacitors and batteries reported in [18,19] have been taken into consideration. In addition, the behavior of the self-discharge voltage curve has been taken into account, which has a voltage drop rate and subsequently stable behavior (see Figure 2). Thus, the proposed model for the self-discharge voltage is Verhulst’s equation given by:

(7) $$\begin{array}{c} \frac{dV}{dt}=r\cdot V\cdot \left(1-\frac{V}{K}\right) \end{array}$$

where *r* is the rate of voltage drop (self-discharge voltage). *K* is the voltage that the electrolyzer maintains after the self-discharge phenomenon, [V].

Equation (7) has the analytical solution:

(8) $$\begin{array}{c} V\left(t\right)=\frac{K\cdot V(0)\cdot \exp[r\cdot t]}{K+V(0)\cdot \exp[r\cdot t]} \end{array}$$

Therefore, by considering Equations (1) and (8), the self-discharge voltage model for PEM electrolyzer is expressed as:

(9) $$\begin{array}{c} V\left(t\right)=\left\{\begin{array}{cc} V_{0} & \mathrm{if}t<t_{c},\\ \; & \\ V_{\mathrm{rev}}+i\left(t\right)\cdot R_{\mathrm{mem}}+\eta_{\mathrm{act}}\left(t\right) & \mathrm{if}t_{c}\leq t<t_{\tau },\\ \; & \\ \frac{K\cdot V\left(t_{\tau }\right)\cdot \exp\left[r\cdot t\right]}{K+V\left(t_{\tau }\right)\cdot \exp\left[r\cdot t\right]} & \mathrm{if}t_{\tau }\leq t. \end{array}\right. \end{array}$$

where $t_{\tau }$ indicates the time in which the self-discharge phenomenon begins in the PEM electrolyzer, [s]. The following section presents the validation and a discussion on the model developed in this section.

## 4. Validation and Discussion

To validate the model, the parameters of Equation (9) have been estimated using an m-file in Matlab*®* together with the command *lsqcurvefit*, which is based on the least-square regression algorithm. The values of the parameters are shown in Table 3.

As can be seen in Table 3, the values of $\eta_{\mathrm{act},c}\left(0\right)$ and $\eta_{\mathrm{act},a}\left(0\right)$ depend on the current input *i*, which agrees with the work reported in [14,40].

After calculating the parameters, the effectiveness of the model at reproducing the real behavior of the self-discharge voltage in the PEM electrolyzer has been evaluated. To carry out this evaluation, a comparison between the experimental data and the model has been created using the mean absolute percentage error $E_{r}$ and the mean absolute error $E_{m}$, as follows:

(10) $$\begin{array}{c} E_{r}=\left(\frac{100}{N}\right)\cdot \left(\sum_{k=1}^{N}\left|\frac{V_{\exp,k}-V_{\mathrm{sim},k}}{V_{\exp,k}}\right|\right) \end{array}$$

(11) $$\begin{array}{c} E_{m}=\left(\frac{1}{N}\right)\cdot \left(\sum_{k=1}^{N}\left|V_{\exp,k}-V_{\mathrm{sim},k}\right|\right) \end{array}$$

where *N* is the number of experimental data. $V_{\exp,k}$ and $V_{\mathrm{sim},k}$ are the *k* experimental data and *k* simulation data, respectively.

**Figure 7:**
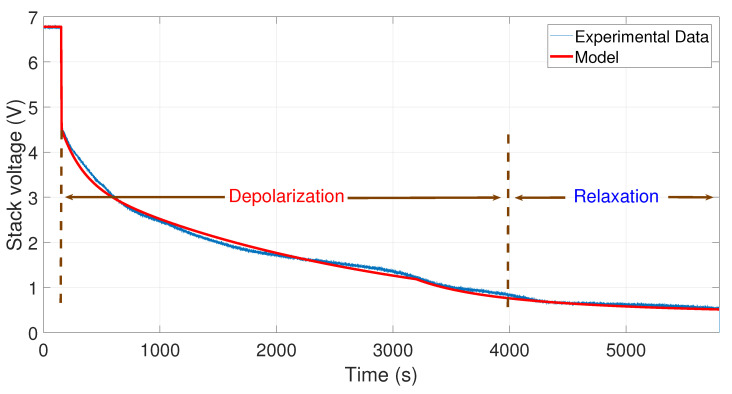
Comparison between the experimental data from 10 to 0 A and the simulation of the model.

**Figure 8:**
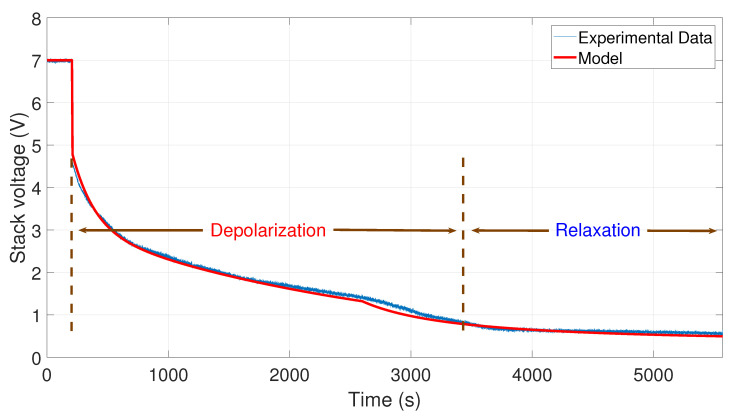
Comparison between the experimental data from 20 to 0 A and the simulation of the model.

**Figure 9:**
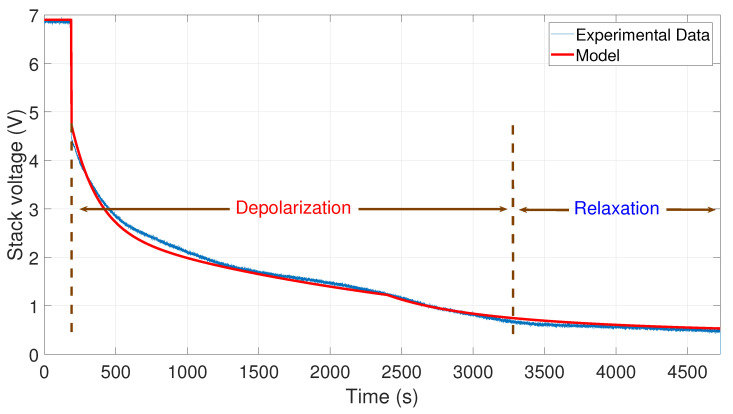
Comparison between the experimental data from 30 to 0 A and the simulation of the model.

**Figure 10:**
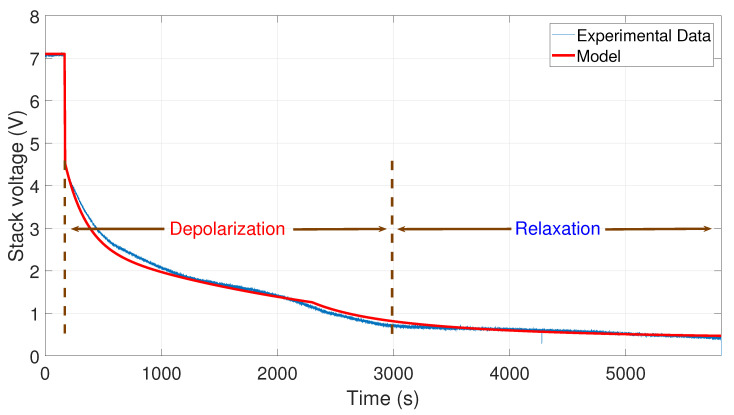
Comparison between the experimental data from 35 to 0 A and the simulation of the model.

To observe the comparison between the experimental data and the model simulations, an m-file was developed in Matlab*®*. The results are shown in Figure 7, Figure 8, Figure 9 and Figure 10. In these figures, the OCV depolarization and relaxation are emphasized. In Figure 7, one can appreciate the comparison between the curve of the experimental data from 10 to 0 A and the curve generated by the model. To generate this simulation, the values $A_{1}=10$ A, $A_{2}=0$ A, $V_{0}=6.78$ V, $t_{c}=155$ s, and $t_{\tau }=3200$ s have been used.

In Figure 8, the comparison between the curve of the experimental data from 20 to 0 A and the curve generated by the model is shown. To perform the simulation, the values $A_{1}=20$ A, $A_{2}=0$ A, $V_{0}=7$ V, $t_{c}=210$ s, and $t_{\tau }=2600$ s have been used.

In Figure 9, one can observe the comparison between the curve of the experimental data from 30 to 0 A and the curve generated by the model. To develop this simulation, the values $A_{1}=30$ A, $A_{2}=0$ A, $V_{0}=6.9$ V, $t_{c}=190$ s, and $t_{\tau }=2400$ s have been used.

Finally, in Figure 10, the comparison between the curve of the experimental data from 35 to 0 A and the curve generated by the model is shown. To perform the simulation, the values $A_{1}=35$ A, $A_{2}=0$ A, $V_{0}=7.1$ V, $t_{c}=170$ s, and $t_{\tau }=2300$ s have been used.

### 4.1. Discussion

To facilitate the comparison between the experimental data and the simulated data, a summary of the relative error and mean error is presented in Table 4. On the one hand, a relative error $E_{r}$ of less than 5% is presented for the tests of 10–0, 20–0, and 30–0 A. The test with 35–0 A obtained a relative error of 5.69%, this being the highest relative error obtained within these comparisons. On the other hand, a mean error $E_{m}$ of less than 0.075 V was obtained for each one of the experimental tests. Therefore, in general, there was an average relative error of $E_{r}=4.6$% and an average mean error of $E_{m}=0.0603$ V for the experimental tests, which validates the model.

As can be seen, the behavior of the self-discharge voltage curve was expected to be asymptotically stable for large periods. Furthermore, the behavior presented by the self-discharge voltage in a PEM electrolyzer has great similarity with the curve described by Verhulst’s equation. Besides, as highlighted in [41], the self-discharge phenomenon in supercapacitors does not obey the usual empirical equations. For this reason, the developed model was based on physical reasoning, taking into consideration the real physical phenomena governed by supercapacitors’ and electrolyzers’ operation. As a result, the model achieved high accuracy.

Finally, recommendations to prevent the phenomenon of self-discharge voltage in a PEM electrolyzer are presented in the next subsection as a result of the analysis of the experimental data.

### 4.2. Self-Discharge Prevention

**Figure 11:**
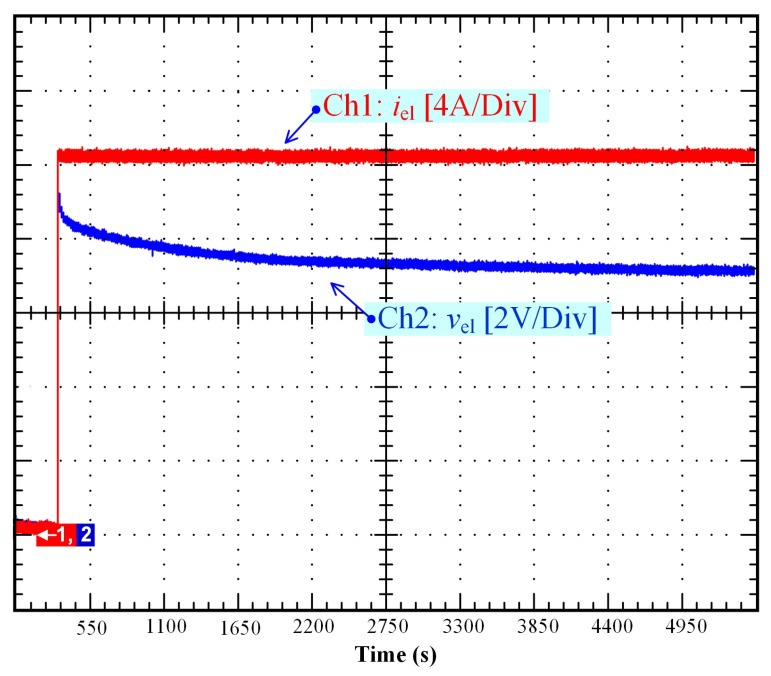
Stack voltage overshoot when connecting to the DC power supply as a result of a self-discharge.

Over the last few years, research has been intensified to develop different solutions to reduce the self-discharging of supercapacitors by providing improvements to the electrodes, separators, or electrolytes [42,43]. Indeed, as highlighted in Section 2, the self-discharge of supercapacitors is an important issue due to rapid self-discharging, low energy efficiency, and loss of stored energy. Like supercapacitors, self-discharge in PEM electrolyzers is a major concern given that gas crossover through the membrane and leakage currents may decrease the lifespan of the electrolyzer and lead to the degradation of the membrane (e.g., pinholes), as reported for PEM fuel cells [32,34]. Besides, when the electrolyzer is reconnected to a power supply with low OCV (as a result of a self-discharge), high overshoot may occur, likely causing deterioration of the PEM electrolyzer [44]. In Figure 11, a voltage overshoot (around 9 V, 12.5% higher than the rated voltage of the electrolyzer (i.e., 8 V)) is shown from when reconnecting the DC power supply (current step from 0 to 20 A). At the beginning of the test, it can be highlighted that the OCV was very low (close to 0 V) as a result of the self-discharge. To prevent the self-discharge issue in PEM electrolyzers, several potential solutions can be adopted:

1. A compromise must be found in the thickness of the membrane to reduce the self-discharging. A thin membrane leads to lower resistance, consequently increasing the gas crossover and leakage currents. An increase of the membrane thickness (improving the permeability of the membrane against gas crossover) leads to higher losses in the membrane, and as a result a decrease in energy efficiency [15,35].
2. The operating temperature must be as low as possible to enhance the protective function of the membrane against gas crossover. A higher operating temperature leads to lower resistance and consequently contributes to gas crossover, as highlighted in previous works focused on the effect of the temperature on Faraday’s efficiency [11].
3. The operating pressure must be as small as possible to limit the gas crossover.
4. To avoid the limitation of the operating conditions (pressure, temperature) while keeping a thinner membrane, the self-discharge can be compensated by supplying the PEM electrolyzer with a small current (contributing to a constant OCV).

## 5. Conclusions

The main objective of this work consisted of introducing to the research field the phenomenon of self-discharge voltage that occurs in a PEM electrolyzer. Additionally, to predict the behavior of the self-discharge voltage, an accurate model has been developed. This model has been developed based on models that have already been reported in the literature for supercapacitors and batteries (i.e., due to the similarities that these devices have with PEM electrolyzers).

The model presented in this work has been validated for self-discharge voltage in a PEM electrolyzer. Besides, this model presents different characteristics and can be useful in different investigations. Indeed, the model has been developed based on physical reasoning, taking into consideration the real physical phenomena governing a PEM electrolyzer’s operation. As a result, this model performs well. However, the model presented in this work can be improved using different mathematical tools, such as functional analysis or control theory. Thus, this work is a basis for future research on the development of more complex models of PEM electrolyzers.

From the analysis performed on the experimental data, recommendations for lowering self-discharge voltage in a PEM electrolyzer have been proposed, taking into consideration many variables, such as temperature, membrane, and pressure.

## Figures and Tables

**Table 1:** Specifications of the HELIOCENTRIS*®* NMH2 1000 PEM electrolyzer.

Parameter	Value	Unit
Rated electrical power	400	W
Stack voltage operating range	4.2–8	V
Stack current range	0–50	A
Operating temperature range	288.15–313.15	K
Hydrogen outlet pressure	10.5	bar
Cells number	3	-
Active area section	50	${\mathrm{cm}}^{2}$
Hydrogen flow rate range at STP (Standard Temperature and Pressure, 20 ${}^{\circ }$C and 1 bar)	0–1	SLPM (Standard Liter Per Minute)

**Table 2:** Summary of the measured duration of the OCV depolarization.

Test	Length of the OCV Depolarization
10–0 A (Figure 3)	3950 s
20–0 A (Figure 4)	3350 s
30–0 A (Figure 5)	3150 s
35–0 A (Figure 6)	2850 s

**Table 3:** Calculated parameters for self-discharge voltage model.

Parameter	Value	Unit
$V_{rev}$	0.21	V
$C_{c}$	800	F
$C_{a}$	800	F
$\tau_{c}$	180	s
$\tau_{a}$	2558.9	s
$\eta_{act,c}\left(0\right)$	5.5719$\cdot \log\left[9.1933\cdot \left(\frac{1}{i}\right)\right]$	V
$\eta_{act,a}\left(0\right)$	$-8.2919\times 10^{-6}\cdot i^{4}+9.8489\times 10^{-4}\cdot i^{3}-0.0314\cdot i^{2}-0.0027\cdot i+3.6606$	V
*r*	−4.1226 $\times \;10^{-4}$	(-)
*K*	0.3941	V

**Table 4:** Summary of the relative and mean errors.

Input Current	$E_{r}$	$E_{m}$
10–0 A	3.55%	0.0528 V
20–0 A	4.43%	0.0489 V
30–0 A	4.70%	0.0745 V
35–0 A	5.69%	0.0648 V

## Data Availability

The data presented in this study are available on request from the corresponding author. The data are not publicly available due to their current utilization for future works involving the authors of this paper.

## Abbreviations

The following abbreviations are used in this manuscript:

OCV	Open-Circuit Voltage
PEM	Proton Exchange Membrane
RES	Renewable Energies Sources
SLPM	Standard Liter Per Minute
SO	Solid Oxide
